# HMB45 protein expression and the immunohistochemical maturation in common blue nevi: a reappraisal^☆^

**DOI:** 10.1016/j.abd.2020.12.013

**Published:** 2022-03-07

**Authors:** Mahmoud Rezk Abdelwahed Hussein

**Affiliations:** Department of Pathology, Assiut University Hospitals, Assiut University, Egypt

**Keywords:** HMB45, Melanocytes, Blue nevi

Dear Editor,

Common blue nevi, first described by Jadassohn-Tièche in 1906, are melanocytic proliferations composed of pigmented cells reminiscent of the embryonal neural crest-derived dendritic melanocytic precursors. They commonly occur on the dorsal aspects of extremities, scalp, and buttocks of children and young adults. Histologically, blue nevi are composed of a symmetrical circumscribed dermal proliferation of pigmented dermal spindle-shaped dendritic melanocytes that have a propensity to extend about the adnexal structures or neurovascular bundles. These dendritic cells are admixed with variable numbers of oval melanocytes reminiscent of intermediate (type B) and spindly neurotized (type C) nevic cells. Common blue nevi arise following the proliferation of the residual dermal dendritic melanocytic pool originating from the neural crest, possibly initiated by dermal inflammation or other stimuli. These nevi may arise from mutated precursor stem cells in the dermis, capable of differentiating into blue nevi.^1^, ^2^

The human homolog of the mouse silver protein (gp100 or Pmel17) is a melanocytes specific type I membrane protein. It is important for the formation of melanosomal fibrils that help the maturation of stage I pre-melanosomes to stage II. The HMB45 antibody was developed from an extract of a lymph-node metastasis of melanoma. It specifically reacts with the glycosylated form of gp100 restricted to the fibrillar matrix of stage II pre-melanosomes.^3^ The gene corresponding to the HMB45 defined protein has recently been cloned and designated gp 100-cl. HMB-45 binds to stage 1 and 2 melanosomes and to the non-melanized portion of stage 3, whereas stage 4 melanosomes and melanosome complexes found in macrophages and keratinocytes have been negative.^3^, ^4^, ^5^ HMB-45 immunoreactivity is frequently noted in fetal and neonatal melanocytes (oncofetal protein) but not in adult resting melanocytes. HMB45 is re-expressed in most malignant melanomas, in the activated melanocytes such as some junctional nevic cells, atypical nevi, traumatized nevus, and melanocytes of blue nevi.^3^, ^5^

To date, knowledge about the expression patterns of HMB45 protein in common blue nevi is limited.^6^, ^7^ The purpose of this study was to examine the maturation of the dermal melanocytes, using HMB45 protein expression, in a series of common blue nevi. To accomplish this goal, the formalin-fixed, paraffin-embedded specimens of common blue nevi (20 cases) and intradermal nevi (20 cases) were retrieved from the archives of the Pathology Department, and the consultation files of this author, at Assuit University Hospitals. The immunohistological sections were reviewed for the expression of the melanocytic (S100: clone 4C4.9, Melan A: clone: A103, HMB45: clone HMB45 monoclonal antibodies) and proliferation markers (Ki67: clone 30-9 polyclonal antibody). The number of HMB45 positive cells (cytoplasmic staining) was evaluated in 100 cells in 4 high-power fields and reported as the percentage of positive cells following other groups.^8^ The data were analyzed using SPSS (version 17, IL, Chicago).

The patients with blue nevi included 11 females and nine males. Their mean age was 53.3 ± 4.1 years (age range: 7‒88 years). The locations with the highest frequencies of blue nevi were the head and neck (nose, forehead, and scalp: 9 cases), extremities (shoulder region and arm:7 cases), and back (2 cases) and chest (2 cases). Most of the cases were pigmented with a clinical appearance similar to seborrheic keratosis or atypical nevi. All common blue and intradermal benign nevi showed strong and diffuse S100 and Melan-A staining throughout the entire lesions. No appreciable KI67 staining was seen in blue or intradermal nevi (Ki67 labeling index was 0‒1%). All cases of intradermal nevi lacked the expression of HMB45 protein. In contrast, all blue nevi expressed HMB45 protein. Three expression patterns were observed: diffuse, patchy, or occasional HMB45 positive cells (Figs. 1‒2). The percentage of positive cells ranged from 5% to 85% (mean value: 49.2 ± 6.4). There was no correlation between the HMB45 expression values and clinical features (age and gender of the patient, or site, size of the lesions).

**Figure 1:**
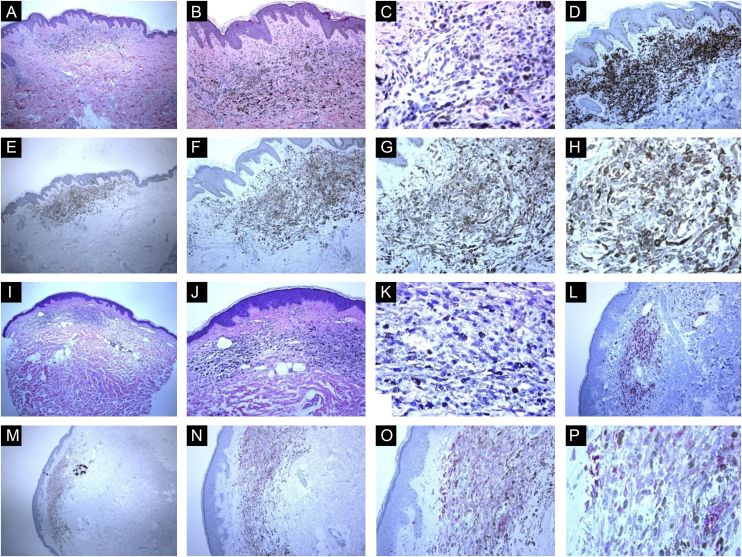
Immunohistological features of common blue nevi with HMB45 protein expression (diffuse or patchy staining patterns). (A, B and C), A case of 59-years old lady presented with 0.5 cm hyperpigmented glistening macule on the skin of the right arm. Histologically, the lesion is composed of a proliferation of spindle-shaped and dendritic melanocytes, admixed with pigment-laden macrophages (“melanophages”). Mitotic figures and junctional melanocytic activity are absent. Histologic maturation is seen at the peripheral and deep parts of the lesion, where the spindle-shaped cells insinuate themselves singly among the thickened collagen fibers of the reticular dermis. A sparse perivascular lymphocytic infiltrate is also seen. There is no individual cell necrosis or cells in mitosis. (D), Further immunohistochemical evaluation was performed with the proper positive and negative controls that revealed strong diffuse Melan-A staining. (E, F, G and H), The tumor cells show HMB45 protein expression throughout the entire lesion (diffuse pattern of staining). (I, J, K and L), A case of a 63-year-old lady with a 0.4 cm hyperpigmented macule on the chest wall. Sections show an admixture of the dendritic epithelioid melanocytes, melanophages in the mid reticular dermis amidst collagen bundles. (L), There is no mitosis or individual cell necrosis or inflammatory cell infiltrate: Further immunohistochemical evaluation was performed with the proper positive and negative controls that revealed strong diffuse Melan-A staining. (M, N, O and P), The tumor cells show diffuse HMB45 protein expression throughout the entire lesion.

**Figure 2:**
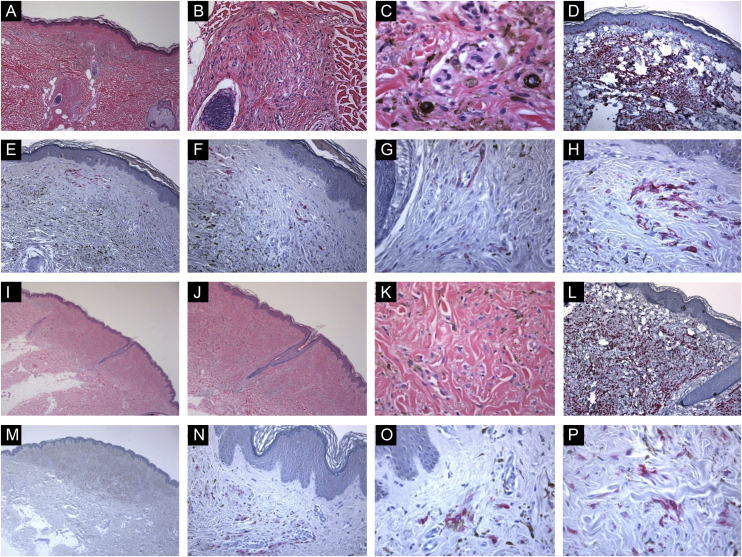
Immunohistological features of common blue nevi with HMB45 protein expression (patchy or individual cell staining patterns). (A, B and C), A case of a 56-year-old lady with a hyperpigmented macule, 0.2 cm, over the upper mid-back. Histologically, there is a well-circumscribed, symmetric dermal growth composed of elongated, finely branching melanocytes insinuated between the collagen fibers of the upper and mid dermis. The melanocytes are admixed with some melanophages. There is periadnexal (perifollicular) aggregation of the melanocytes. (D), Further immunohistochemical evaluation was performed with the proper positive and negative controls that revealed strong diffuse Melan-A staining throughout the entire lesion. (E, F, G and H), Some groups of HMB45 positive dermal melanocytes are noted (patchy pattern of staining). (I, J, K and L), A case of a 49-year-old lady with a smooth, gray-tan lesion, 0.7 cm in size, over the skin of the left buttock. Histological sections show alteration of the dermis by a symmetric growth consisting of a variable admixture of dendritic melanocytes, some melanophages, and fibrosis. There is no apparent cytologic atypia, cell necrosis, or mitotic activity. Further immunohistochemical evaluation was performed with the proper positive and negative controls that revealed strong diffuse Melan-A staining throughout the entire lesion. (M, N, O and P), Occasional HMB45 positive dermal nevic cells are noted (individual-cell pattern of staining).

Several studies indicated that HMB-45 staining could highlight the pattern of “immunohistochemical maturation” of the melanocytic lesions. The activated junctional or superficial, type A melanocytes (epithelioid cells) express HMB45, while the deeply located type C melanocytes (spindle cells) do not express this antibody.^3^, ^5^ In agreement with the previous investigations,^6^ all common blue nevi in this study showed a variable expression of HMB45 proteins. Wood et al. immunohistochemically examined HMB45 protein expression in blue nevi. They found diffuse strong HMB45 reactivity in all cellular blue nevi and variable HMB45 protein expression in most of the common blue nevi. The authors suggested that blue nevi have an activated phenotype. Similarly, Sun et al reported the expression of HMB45 protein in 16 cases of blue nevi. The authors suggested that the nevic cells of blue nevi cells are derived from a precursor cell that has some common features of both melanocyte and Schwann cells.^7^

In this study, the preserved HMB45 protein expression in both superficial and deep portions of the lesions implies the lack of immunohistochemical maturation in blue nevi. This preserved HMB45 protein expression in these nevic cells is indicative of their activated phenotype with active melanosome formation. Although mechanisms underlying this activated phenotype in common blue nevi are unknown, it may be reasoned to the release of melanocytic growth factors such as hepatocyte growth factor, endothelin-1, and α-melanocyte-stimulating hormone. These factors can alter the HMB45 protein glycosylation during various pathogenic states of melanocytes, help activate melanogenesis, and stimulate the motility and proliferation of the normal resting residual cells at the dermal melanocyte pools.^9^ It is possible that the cells of the common blue nevi directly arise from the activated extrafollicular dermal melanocyte stem cells. The latter persist after birth in the superficial nerve sheath of peripheral nerves. These cells can proliferate, giving rise to migratory melanocyte precursors with activated phenotype and HMB45 protein expression.^10^ To conclude, this study reported HMB45 protein expression in the dermal melanocytes of the common blue nevi, indicative of their activated phenotype. The underlying mechanisms of this activated phenotype, such as the role of growth factors, are open for further investigations.

## Financial support

None declared.

## Authors’ contributions

Mahmoud Rezk Abbelwahed Hussein fully and solely contributed to the followings: Approval of the final version of the manuscript; critical literature review; data collection, analysis, and interpretation; effective participation in research orientation; intellectual participation in propaedeutic and/or therapeutic; management of studied cases; manuscript critical review; preparation and writing of the manuscript; statistical analysis; and study conception and planning.

## Conflicts of interest

None declared.
